# *In Vivo* Antimalarial Activity of Leaf Latex of *Aloe melanacantha* against *Plasmodium berghei* Infected Mice

**DOI:** 10.1155/2021/6690725

**Published:** 2021-06-21

**Authors:** Gebrehiwot Kiros Gebremariam, Haile Kassahun Desta, Tekleab Teka Teklehaimanot, Tsgab Gebrecherkos Girmay

**Affiliations:** ^1^Department of Biology, College of Natural and Computational Science, Aksum University, Aksum, Ethiopia; ^2^Department of Pharmacy, College of Medicine and Health Science, Wollo University, Dessie, Ethiopia; ^3^Department of Statistics, College of Natural and Computational Science, Aksum University, Aksum, Ethiopia

## Abstract

**Background:**

Malaria is a major health concern in the world in general and developing countries in particular. Nowadays, the control of malaria has ended up steadily more complex due to the spread of drug-resistant parasites. Medicinal plants are the verifiable source of compelling antimalarial drugs. The present study was aimed to assess the *in vivo* antimalarial activity of leaf latex of *A. melanacantha* against *Plasmodium berghei* in mice.

**Methods:**

Acute oral toxicity study of the leaf latex was assessed in mice up to a dose of 2,000 mg/kg. A four-day suppressive model was utilized to investigate the antimalarial activity of the plant. Three extract doses, 100, 200, and 400 mg/kg/day, doses of the plant leaf latex, chloroquine, 10 mg/kg (positive control) and distilled water, and 10 mL/kg (negative control) were administered to mice. Percent parasitemia suppression, packed cell volume, mean survival time, body weight, and rectal body temperature were used to determine antimalarial activity.

**Results:**

Test groups treated with 100, 200, and 400 mg/kg of the latex showed a significant parasitemia suppression in dose dependent manner compared to the negative control with an IC_50_ of 22.63 mg/ml. Mice treated with 100, 200, and 400 mg/kg have shown parasitemia suppression of 14.86%, 29%, and 43.2%, respectively. The chemosuppression was significant (*P* < 0.05) at all doses compared to the negative control. Similarly, mice treated with 100 mg/kg, 200 mg/kg, and 400 mg/kg have shown a significant survival time compared to the negative control. At the same time, weight loss reduction was observed within the test groups treated with 100 mg/kg and 200 mg/kg of the latex while the test groups treated with 400 mg/kg had showed almost no weight loss reduction. The latex also reversed the PCV reduction significantly (*P* < 0.05) at 200 mg/kg and 400 mg/kg doses and prevented rectal temperature dropping significantly (*P* < 0.05) at all doses.

**Conclusion:**

The leaf latex of *A. melanacantha* has shown significant antimalarial activity against *P. berghei* in mice supporting the genuine traditional antimalarial usage of the plant.

## 1. Introduction

Malaria is a major health concern in the world in general and developing countries in particular. It is the major cause of mortality and dismalness within the malaria-endemic nations. Indeed although the dissemination of the disease is considerably shifted, sub-Saharan Africa, Asia, and Central and Latin America are the foremost affected regions [1]. It has been estimated that almost 50% of the population within the world live in a malaria-risk area. In 2016, an assessed 216 million cases of malaria happened around the world and there were an assessed 445,000 deaths from malaria globally. The burden of the disease is the heaviest in Africa, where 82 and 90% of all worldwide cases and deaths happened, respectively [2].

Despite progress in advanced medicine, malaria remains a disease troublesome to eradicate, in this manner a major health issue. According to WHO, 2017 report, despite these reductions between 2014 and 2016, considerable increments in case rates happened in the Americas, South-East Asia, Western Pacific, and African regions. Particularly, fifteen nations accounted for 80% of worldwide malaria deaths in 2016; all of these nations are in sub-Saharan Africa.

In Ethiopia, even though a few advancements were as of late accomplished, malaria is still the driving cause of morbidity and mortality. Ethiopia is additionally one of the foremost malaria epidemic-prone nations in Africa where rates of morbidity and mortality increase 3–5-folds amid epidemics. For example, together with India, Indonesia, and Pakistan, Ethiopia accounts for about 75% and 84% cases and deaths due to *P. vivax,* respectively [3]. Resistance of mosquitoes to bug sprays and antimalarial drugs particularly to the current most viable and most up to date artemisinin subsidiaries have been complicated the eradication of the disease [4]. These repeating issues render the advancement and promotion of phytomedicines as an elective solution to malaria control. Medicinal plants have been playing an imperative part in the treatment of malaria for centuries and have continuously been considered to be a conceivable alternative and rich source of new drugs [5]. Nowadays, home grown items are being utilized around the world in an assortment of healthcare settings, and as domestic cures [6]. Over 1200 plant species from 160 families are utilized to treat malaria and fever in endemic nations [4]. It is almost evaluated that about 80% of the population in Ethiopia is still dependent on traditional medicines. However, scientific studies on the status of utilization of phytomedicine, preparation of crude extracts, and isolation of active compounds are very minimal.

Aloe species latex is specifically used for the treatment of parasitic disease including malaria, fungal infections, bacterial infections, gastrointestinal disorders, and inflammatory diseases [7]. The leaf latex of *A. melanacantha* has been used traditionally for the treatment of malaria and wound in Ethiopia [8]. The traditional use of *Aloe melanacantha* for malaria treatment is not however scientifically approved. Thus, the present study was conducted to assess the antimalarial activity of leaf latex of *A. melanacantha* using a 4-day suppressive test.

## 2. Materials and Methods

### 2.1. Collection of Plant Material

The leaf latex of *Aloe melanacantha* was gathered from the periphery of Ataye town, 60 km away from Dessie city to Addis Ababa, Eastern Ethiopia. Confirmation of the plant material was done by Professor Sebsebe Demissew, Department of Biology, Addis Ababa University, and it was given a voucher specimen GK001 which was kept in national herbarium of Addis Ababa University.

### 2.2. Instruments, Drugs, Reagents, and Chemicals

The following listed drugs, reagents, and chemicals were utilized to conduct the experiments: chloroquine (Addis Pharmaceuticals Factory, Ethiopia), trisodium citrate (Deluxe Scientific Surgical, India), Giemsa (Science lab, USA), ketamine (Rotex medica, Germany), and normal saline (Addis Pharmaceuticals Factory, Ethiopia), HCl, water bath, Mayer's reagent, benzene, Tween 80, ethyl acetate, ammonium solution, Aluminum chloride solution, sulphuric acid, NaOH, and ferric chloride solution were utilized. All reagents were systematically reviewed and obtained from certified providers. Test tubes, Beaker, Whatmann filter, graduated cylinder, Stopwatch, oral gavages, digital thermometer, electronic weighing balance, and compound microscope (Olympus, Germany) were also utilized to conduct the study.

### 2.3. Preparation of Plant Extract

The leaves of *A. melanacantha* were cut transversally near the base and then inclined in a plastic material to collect the yellow sap from inside of the leaf. The collected sap was then left in shaded open air for two to three days in a wider plastic material by making a thin film which was dried through evaporation to give pale yellow latex. A total of 10.5 g leaf latex extract was yielded. The dried latex was powdered using electric grinding mill and made ready for the study [9, 10].

### 2.4. Experimental Animals

Male Swiss albino mice weighing 18–25 g with 6–8 weeks of age were used to study the antimalarial activity of the latex while Swiss albino mice of female sex weighing 25–30 g with 8–12 weeks of age were used to conduct the acute oral toxicity study [11, 12]. All the animals were obtained from the animal house of the Department of Pharmacy, Wollo University, Ethiopia. They were kept up at a temperature of 22 ± 2°C with 12 hr light and 12 hr dark cycle and given nourishment and water. The mice were acclimatized to the experimental environment one week earlier to the study [6]. They were utilized as per the international law for the care and utilization of experimental animals.

### 2.5. Parasites

To conduct the study chloroquine sensitive *Plasmodium berghei* ANKA strain was utilized. The strain was obtained from the Ethiopian Public Health Institute (EPHI), Addis Ababa, Ethiopia, and kept up by a serial section of blood from infected mice to noninfected ones on weekly basis.

### 2.6. Phytochemical Screening

The leaf latex of *Aloe melanacantha* was screened to ascertain the presence of different secondary metabolites following the standard procedures depicted somewhere else [13].

### 2.7. Acute Oral Toxicity Study

Nulliparous nonpregnant female Swiss albino mice were utilized to conduct the acute oral toxicity of the latex. A total of five healthy mice were used and they were having access to tap water and food, except for an overnight fasting period (12 h) before oral administration of a single dose of the sample [10]. The mice were weighed and test doses/samples of the latex, calculated in connection to the bodyweight of each fasted mouse, were dissolved in distilled water and administered via oral gavage at 2000 mg/kg. The mice were then routinely and separately watched for behavioral changes and common toxicity signs after dosing for the primary 24 h with extraordinary attention being given amid the primary 4 hrs. The mice were further observed for up to 14 days following treatment for any signs of gross behavioral changes such as altering of feeding, lacrimation, hair erection, mortality, and other signs of toxicity appearance and the latency of death [11].

### 2.8. Grouping and Dosing of Animals

The experimental study design was utilized, and a simple random sampling technique was employed for the grouping of experimental animals and assignments of treatments [4]. A total of 25 mice inoculated with *Plasmodium berghei* were arbitrarily assigned into five groups with five mice in each group. The leaf latex of *A. melanacantha* was prepared in three doses by dissolving in distilled water. Three hours after infection, group I of mice were treated with the vehicle (distilled water, 10 ml/kg, which served as a negative control), groups II, III, and IV of mice were treated with 100, 200, and 400 mg/kg of the latex, respectively, which served as test groups, and group V of mice were treated with a standard drug chloroquine (Addis Pharmaceuticals Factory, Ethiopia) 10 mg/kg which served as positive control. All treatments were administered safely to mice via an intragastric route using oral gavages and repeated for the next 3 days (D1 to D3). The mice received the drug for 4 days [4, 14].

### 2.9. In Vivo Antimalarial Activity Test

Swiss albino mice infected with *P. berghei* and having a parasitemia level of 20–30% were utilized as a donor. The benefactor mice would be at that point sacrificed by execution and blood was collected by cardiac cut into a heparinized tube containing 0.5% trisodium citrate. The blood was then diluted with physiological saline (0.9%) based on parasitemia level of the giver mice and the red blood cell (RBC) tally of typical mice, in such a way that 1 ml blood contains 5 × 10^7^ contaminated RBCs. Each mouse was then given 0.2 ml of this diluted blood intraperitoneally, which contained 1 × 10^7^*Plasmodium berghei* infected RBCs [5].

The *in vivo* antimalarial activity was assessed by the 4-day chemosuppressive standard test portrayed by Peters and Robinson [15]. After standard parasite inoculation, 25 mice were arbitrarily partitioned into five groups of 5 mice each and faceted for three hours. Treatments were then given and preceded for three sequential days (D0 to D3). On the fifth day (D4), the following parameters were actualized to decide the in vivo antimalarial activity of the latex.

Thin blood smears were prepared from the tail of each mouse and applied on microscopic slides (Science lab, USA) and the blood was drawn equitably over a second slide to make thin blood film and allowed to dry at room temperature. They were fixed with absolute methanol and stained with 10% Giemsa stain for 20 minutes. Each slide was then studied under a compound microscope and data obtained were recorded and analyzed to determine percent parasitemia. The parasitemia was decided by checking the least of three areas per slide with 100 RBC per field. The smears were studied and counted by a laboratory technician to make the reader blind. Average percent parasitemia and suppression were then determined using the following formula [15–17]:

(1) $$\begin{array}{c} \text{\% parasitemia}=\frac{\text{number of parasitized RBC}}{\text{total number of RBC}}\times 100,\\ \text{\% suppression}=\frac{\text{mean parasitemia of NC}-\text{mean parasitemia of TG}}{\text{mean parasitemia of NC}}\times 100, \end{array}$$

where NC is negative control and TG is treated group.

The body weight of each mouse in all groups was measured before infection (day 0) and after infection (day 4) using an electronic balance. The rectal temperature of each mouse was also measured with a digital thermometer before infection, three hours after infection, and then daily up to day 4 to see the impact of the latex on body temperature [16, 18, 19].

Packed cell volume (PCV) was measured by taking blood from the tail of each mouse, using heparinized microhematocrit capillary tubes. The tubes were fixed by the precious stone seal and set in a microhematocrit centrifuge (Hawksley, Spearing, UK) with the fixed close outwards. The blood was then centrifuged at 12,000 revolutions per minute for 5 minutes. The packed cell volume (PCV) of each mouse was measured before infection and after infection using the following formula [20]:

(2) $$\begin{array}{c} \text{PCV}=\frac{\text{volume of erythrocytes in a given volume of blood}}{\text{total blood volume}}. \end{array}$$

Moreover, the mean survival time (MST) was utilized to assess the efficacy of the antimalarial plant extract. Mortality was checked every day and the number of days from the time of inoculation of the parasite up to death was recorded for each mouse within the treatment and control groups all through the follow-up period. The survival time for each mouse was recorded after the treatment periods and the mean survival time for each group was then calculated as follows [19, 21]:

(3) $$\begin{array}{c} \text{MST}=\frac{\text{sum of survival time of mice in a group}\left(\text{Days}\right)}{\text{total numbers of mice in that group}}. \end{array}$$

### 2.10. Ethical Clearance

This study was approved by the ethical review committee of Wollo University, College of Medicine and Health Sciences, Ethiopia (protocol number WU Phar/489/2010). The experiment was done in accordance with the guide for the care and use of laboratory animals [22].

### 2.11. Data Analysis

Results of parasitemia, changes in survival time, body weight, and packed cell volume were analyzed utilizing SPSS software version 20 and detailed as mean ± standard error (mean ± SEM) for each treatment group. One way ANOVA and Tukey post hoc tests were utilized to analyze differences between groups and subgroups/within groups/respectively. Differences were considered as statistically significant in case *P* value <0.05.

## 3. Results

### 3.1. Phytochemical Screening

Phytochemical screening of the latex revealed the presence of anthraquinones, flavonoid, glycoside, saponins, tannins, and terpenoids, and the absence of alkaloids (Table 1).

### 3.2. *In Vivo* Acute Oral Toxicity of the Latex

The acute toxicity test showed that the leaf latex of *A. melanacantha* caused neither mortality within the first 24 hrs nor obvious signs of acute toxicity such as tremor, lacrimation, hair erection, the misfortune of craving, salivation, and diarrhea within the following 14 days at the dosage used (2000 mg/kg).

### 3.3. *In Vivo* Antimalarial Activity Test

#### 3.3.1. Four-Day Chemosuppressive Antimalarial Activity of the Latex

The leaves latex of *Aloe melanacantha* has shown significant antimalarial activity against *Plasmodium berghei* infected mice. The four-day suppressive test result showed that the leaf latex of *A. melanacantha* has showed parasitemia suppression in dose dependent manner. Mice treated with 100, 200, and 400 mg/kg have shown parasitemia suppression of 14.86%, 29%, and 43.2%, respectively. The chemosuppression was significant (*P* < 0.05) at all doses compared to the negative control (Table 2). IC_50_ of the antimalarial activity of this medicinal plant was 22.63 mg/ml. The leaf latex of *A. melanacantha* showed better survival time while mice in the negative control died before 7 days. Mice treated with 100, 200, and 400 mg/kg survived for significantly (*P* < 0.05) longer time as compared to the negative control mice (Table 2).

The leaf latex of *A. melanacantha* has prevented weight loss reduction in dose independent manner. Compared to the negative control, significant weight loss reduction was observed with the mice treated with 100 mg/kg (*P* < 0.05) and 200 mg/kg of the latex (*P* < 0.05) while mice treated with 400 mg/kg have not showed significant weight loss reduction (Table 3).

Analysis of the rectal temperature of infected mice also showed that all the test groups treated with the latex had no rapid dropping of rectal temperature significantly (*P* < 0.05) compared to the negative control (Table 4). Moreover, the leaf latex of *A. melanacantha* prevented the reduction of packed cell volume significantly (*P* < 0.05) at 200 mg/kg and 400 mg/kg doses compared to the negative control while the lowest dose (100 mg/kg) failed to significantly reverse PCV reduction (Table 5).

## 4. Discussion

About 80% of the population of the world still depend intensely on traditional healers and require medicinal plants for the treatment of different sorts of sicknesses. Traditional medications are regularly more accessible, reasonable, and now and then are seen as more viable than advanced drugs. Ethiopia is among the malaria-endemic countries in the world although it is wealthy in flora that may be sources of different, new antimalarial agents [23–25]. Management of malaria is a big challenge due to the increasing number of cases of resistance to the majority of currently available drugs, even to new artemisinin-based combination therapy (ACT) drugs. *P. falciparum* malaria is a life-threatening disease if not treated promptly with effective antimalarial medicines. So, there is an urgent need to find new alternative drugs, which have novel modes of action. Therefore, we made an effort to assess antimalarial activity of the leaf latex of *A. melanacantha* in *P. berghei* infected mice models.

In vivo antimalarial activity study is the foremost broadly utilized method to assess the antimalarial activity of natural and synthetic antimalarial agents. The four-day chemosuppressive method is also the most extensively used method to study the preliminary antimalarial activity of new entities [26]. In the present study, the leaf latex of *A. melanacantha* has shown significant antimalarial activity as compared to the negative control in a dose dependent manner. The highest suppression was observed at the maximum dose given (400 mg/kg). Hence, the present study provides the scientific evidence for the folkloric use of the plant in the treatment of malaria. The antimalarial activity of the latex of *A. melanacantha* could be due to a single compound or synergetic effect of the secondary metabolites found in the plant. Similar findings were reported by previous studies on the leaf latex of *Aloe* species such as *A. pulcherrima, A. debrana, A. percrassa, A. citrana, A. vera,* and *Aloe perryi* which have shown significant antimalarial activities [27].

Survival time is another parameter to evaluate antimalarial activity of plant extracts and if an extract results in a survival time greater than negative control group, it is considered as an active antimalarial agent [27, 28]. The latex of the plant has shown a better survival time compared to the negative control and, hence, the latex is active for antimalarial effects against the *Plasmodium berghei* infected mice. *A. pulcherrima, A. debrana,* and *A. percrassa* have also shown similar results [26].

Body weight loss prevention is also another parameter to confirm the antimalarial activity of new natural or synthetic antimalarial drugs as body weight loss is a characteristic of *Plasmodium berghei* infected mice resulting from appetite loss, metabolic disturbance, and hypoglycemic effect of the parasite [29]. Although an increase in weight was not consistent with an increase in dose, the latex had shown some degree of weight loss prevention at the doses of 100 and 200 mg/kg. But weight loss prevention was low at the highest test dose (400 mg/kg) which could be due to the presence of appetite suppressant secondary metabolite in the latex. Similar findings were reported in previous studies on other medicinal plants [12, 30, 31].

Anemia and reduction in rectal body temperature are also among the general features of *Plasmodium berghei* infected mice. In infected mice, diminish in metabolic rate happens before death and is accompanied by a corresponding decrease in internal body temperature [32]. Rectal temperature decreases as parasite level escalates and active secondary metabolites must prevent the rapid dropping of rectal temperature. Although the leaf latex of *A. melanacantha* failed to prevent parasite induced rectal temperature reduction at the lower test doses of 100 mg/kg and 200 mg/kg, it has shown a significant defensive impact against internal body temperature diminishment at the dose of 400 mg/kg. This protective effect of *A. melanacantha* leaf latex against temperature reduction reflects constituents responsible for this effect were likely found in this latex [20].

PCV is measured to assess the effectiveness of a crude drug in preventing hemolysis due to the rising parasitemia level [31]. The main causes of anemia in mice and human include clearance or destruction of infected RBCs, the clearance of uninfected RBCs, erythropoietic suppression, and dyserythropoiesis. In untreated mice, the parasite count increased, and the hematocrit PCV decreased markedly from day to day until the death of the animal, which was observed in the previous studies [13, 30, 33]. In the current study, the dose of 100 mg/kg has not reversed the PCV reduction significantly as compared to the negative control while 200 mg/kg and 400 mg/kg doses and chloroquine reversed the PCV reduction significantly (*P* < 0.05). This could be due to the antiplasmodial effect of the latex and as a result of sustaining the availability of new RBCs produced in the bone marrow [23, 34].

Based on phytochemical screening, the leaf latex of *A. melanacantha* was found to be positive for the presence of anthraquinones, glycosides, saponins, terpenoids, tannins, and flavonoids which were known to have antimalarial activity. Hence, the antimalarial activity of the plant might be due to their secondary metabolites such as anthraquinones, glycosides, saponins, terpenoids, tannins, and flavonoids [35–37]. The antimalarial activity of *Aloe melanacantha* could moreover be due to a single or combined impact of the secondary metabolites found in the latex. The active metabolite/s of the study plant is yet to be isolated and identified. Few of the possible mechanisms for antimalarial activity of the above secondary metabolites are mainly inhibition of protein synthesis, DNA intercalation, antioxidation, and free radical scavenging [9, 38].

## 5. Conclusion

*Aloe melanacantha* has shown significant antimalarial activity against *Plasmodium berghei* infected mice and this confirms the genuine traditional use of the medicinal plant in the rural areas of Ethiopia around Ataye, Northeast Ethiopia. The antimalarial activity of the plant might be related to the presence of secondary metabolites which might act in single or in combination against *Plasmodium berghei* infection. As a result, the plant could be a potential medicinal plant for further discovery of promising antimalarial drugs by isolating and structural elucidation of the secondary metabolites.

## Figures and Tables

**Table 1:** Preliminary phytochemical screening of the leaf latex of *Aloe melanacantha*.

Phytochemical components of qualitative analysis	Leaf latex of *Aloe melanacantha*
Alkaloids	—
Anthraquinones	+++
Flavonoids	++
Glycoside	++
Saponins	++
Tannins	+
Terpenoids	++

+++ = strong presence; ++ medium presence; + = low presence; — = absence.

**Table 2:** Four-day chemosuppression antimalarial activities and effect on survival time of leaf latex of *A. melanacantha* against *Plasmodium berghei* infected mice.

Test substance	Doses (mg/kg)	% parasitemia	% suppression	Survival days
Distilled water	10 ml/kg	29.6 ± 2	0	6.00
*A. melanacantha*	100 mg/kg	25.2 ± 1.92^a^	14.86	8.6 ± 1.67^a^
Leaf latex	200 mg/kg	21 ± 1.4^a^	29	9.8 ± 0.83^a^
	400 mg/kg	16.8 ± 2.38^a^	43.2	8.8 ± 2^a^
Chloroquine	10 mg/kg	0.00^b^	100	ND

Data were expressed as mean ± standard deviation (*n* = 5) where a = significant compared to the control group, b = significant compared to control group, and ND = no death within the follow-up period.

**Table 3:** Effect of *A. melanacantha* leaf latex on body weight of *Plasmodium berghei* infected mice.

Test substance	Doses (mg/kg)	D0	D4	Weight change
Distilled water	10 ml/kg	40.32 ± 2.3	35.4 ± 3.1	−4.92
*A. melanacantha*	100 mg/kg	33.38 ± 0.9	31 ± 2.7	−2.38^a^
Leaf latex	200 mg/kg	34.18 ± 2.28	32.1 ± 2.87	−2.08^a^
	400 mg/kg	41.1 ± 0.92	36.8 ± 2.65	−4.3
Chloroquine	10 mg/kg	40.3 ± 2.3	41.6 ± 2.2	1.3^b^

Data are expressed as mean ± standard deviation (*n* = 5) where a = significant compared to the control group, b = significant compared to control group (CQ), D0 = day 0, and D4 = day 4.

**Table 4:** Effect of *A. melanacantha* leaf latex on body temperature of *Plasmodium berghei* infected mice.

Test substance	Doses (mg/kg)	D0	D4	% Change
Distilled water	10 ml/kg	37.51 ± 0.21	37.3 ± 0.29	−0.21
*A. melanacantha*	100 mg/kg	37.1 ± 0.16	36.97 ± 0.19	−0.13^a^
Leaf latex	200 mg/kg	37 ± 0.5	36.94 ± 0.59	−0.06^a^
	400 mg/kg	37.1 ± 0.42	37.39 ± 0.19	0.09^a^
Chloroquine	10 mg/kg	36.8 ± 0.55	36.86 ± 0.69	0.01^b^

Values are expressed as mean ± standard deviation (*n* = 5), a = significant compared to the control group, b = significant compared to the control group, D0 = day 0, and D4 = day 4.

**Table 5:** Effect of *Aloe melanacantha* leaf latex on packed cell volume of *Plasmodium berghei* infected mice.

Test substance	Doses (mg/kg)	D0	D4	PCV change
Distilled water	10 ml/kg	60.8 ± 0.04	60.4 ± 0.04	−0.4
*A. melanacantha*	100 mg/kg	56.8 ± 0.08	56.2 ± 0.85	−0.6
Leaf latex	200 mg/kg	55 ± 0.06	55.4 ± 0.71	0.4^a^
	400 mg/kg	54.2 ± 0.055	54.4 ± 0.08	0.2^a^
Chloroquine	10 mg/kg	59.8 ± 0.02	60.4 ± 0.02	0.6^b^

Values are expressed as mean ± standard deviation (*n* = 5), a = significant compared to the control group, b = significant compared to control group (CQ), D0 = day 0, and D4 = day 4.

## Data Availability

The data utilized to back the findings of this study are included within the article.

## References

[1] World Health Organization (2015). *World Malaria Report 2014: Summary*.

[2] World Health Organization (2017). *World Malaria Report 2017*.

[3] World Health Organization (2016). *World Malaria Report 2015*.

[4] Muluye A. B., Melese E., Adinew G. M. (2015). Antimalarial activity of 80% methanolic extract of *Brassica nigra* (L.) Koch. (*Brassicaceae*) seeds against *Plasmodium berghei* infection in mice. *BMC Complementary and Alternative Medicine*.

[5] Asnake S., Teklehaymanot T., Hymete A., Erko B., Giday M. (2016). Survey of medicinal plants used to treat malaria by Sidama people of Boricha District, Sidama Zone, South region of Ethiopia. *Evidence-Based Complementary and Alternative Medicine*.

[6] Tesfaye W. H., Alamneh E. A. (2014). In vivo antimalarial activity of the crude extract and solvent fractions of the leaves of *Zehenria scabra* (Cucurbitaceae) against *Plasmodium berghei* in mice. *Journal of Medicinal Plants Research*.

[7] Cock I. E. (2015). The genus aloe: phytochemistry and therapeutic uses including treatments for gastrointestinal conditions and chronic inflammation. *Novel Natural Products: Therapeutic Effects in Pain, Arthritis and Gastro-Intestinal Diseases*.

[8] Teka T. (2018). The leaf latex of *A. melanacantha* has been used traditionally for the treatment of malaria in Ethiopia. *Verbal Communication with Traditional Healer*.

[9] Teka T., Bisrat D., Yeshak M., Asres K. (2016). Antimalarial activity of the chemical constituents of the leaf latex of *Aloe pulcherrima* Gilbert and Sebsebe. *Molecules*.

[10] Teka T., Bisrat D., Mazumder A., Asres K. (2014). Antimicrobial constituents from the leaf latex of *Aloe pulcherima* Gilbert & Sebsebe. *International Journal of Phytopharmacology*.

[11] Guideline O. O. (2001). 425: acute oral toxicity—up-and-down procedure. *OECD Guidelines for the Testing of Chemicals*.

[12] Bello I., Bakkouri A., Tabana Y. (2016). Acute and sub-acute toxicity evaluation of the methanolic extract of *Alstonia scholaris* stem bark. *Medical Sciences*.

[13] Asrade S., Mengesha Y., Moges G., Gelayee D. A. (2017). In vivo antiplasmodial activity evaluation of the leaves of *Balanites rotundifolia* (Van Tiegh.) Blatter (Balanitaceae) against *Plasmodium berghei*. *Journal of Experimental Pharmacology*.

[14] Mzena T., Swai H., Chacha M. (2018). Antimalarial activity of *Cucumis metuliferus* and *Lippia kituiensis* against *Plasmodium berghei* infection in mice. *Research and Reports in Tropical Medicine*.

[15] Peters W., Robinson B. L. (1992). The chemotherapy of rodent malaria. XLVII. Studies on pyronaridine and other Mannich base antimalarials. *Annals of Tropical Medicine & Parasitology*.

[16] Kalra B., Chawla S., Gupta P., Valecha N. (2006). Screening of antimalarial drugs: an overview. *Indian Journal of Pharmacology*.

[17] Krettli A., Adebayo J., Krettli L. (2009). Testing of natural products and synthetic molecules aiming at new antimalarials. *Current Drug Targets*.

[18] Deressa T., Mekonnen Y., Animut A (2010). In vivo anti-malarial activities of *Clerodendrum myricoides*, *Dodonea angustifolia* and *Aloe debrana* against *Plasmodium berghei*. *Ethiopian Journal of Health Development*.

[19] Kumar S., Yadav M., Yadav A., Rohilla P., Yadav J. P. (2017). Antiplasmodial potential and quantification of aloin and aloe-emodin in Aloe vera collected from different climatic regions of India. *BMC Complementary and Alternative Medicine*.

[20] Bantie L., Assefa S., Teklehaimanot T., Engidawork E. (2014). In vivo antimalarial activity of the crude leaf extract and solvent fractions of *Croton macrostachyus* Hocsht. (Euphorbiaceae) against *Plasmodium berghei* in mice. *BMC Complementary and Alternative Medicine*.

[21] Dikasso D., Makonnen E., Debella A. (2006). In vivo anti-malarial activity of hydroalcoholic extracts from *Asparagus africanus* Lam. in mice infected with *Plasmodium berghei*. *Ethiopian Journal of Health Development*.

[22] National Research Council (2010). *Guide for the Care and Use of Laboratory Animals*.

[23] Bihonegn T., Giday M., Yimer G., Animut A., Sisay M. (2019). Antimalarial activity of hydromethanolic extract and its solvent fractions of *Vernonia amygdalina* leaves in mice infected with *Plasmodium berghei*. *SAGE Open Medicine*.

[24] Basir R., Rahiman S. F., Hasballah K. (2012). *Plasmodium berghei* ANKA infection in ICR mice as a model of cerebral malaria. *Iranian Journal of Parasitology*.

[25] Tadesse B., Terefe G., Kebede N., Shibeshi W. (2015). In vivo anti-trypanosomal activity of dichloromethane and methanol crude leaf extracts of *Dovyalis abyssinica* (Salicaceae) against *Trypanosoma congolense*. *BMC Complementary and Alternative Medicine*.

[26] Muluye A. B., Desta A. G., Abate S. K., Dano G. T. (2019). Anti-malarial activity of the root extract of *Euphorbia abyssinica* (*Euphorbiaceae*) against *Plasmodium berghei* infection in mice. *Malaria Journal*.

[27] Alshawsh M. A., Mothana R. A., Al-shamahy H. A., Alsllami S. F., Lindequist U. (2009). Assessment of antimalarial activity against *Plasmodium falciparum* and phytochemical screening of some Yemeni medicinal plants. *Evidence-Based Complementary and Alternative Medicine*.

[28] Mengiste B., Mekuria A., Aleme H., Afera B., Negash G. (2014). Treatment of skin disease using ointment of latex of *Euphorbia abyssinica* medicinal plant on animal model. *World Applied Sciences Journal*.

[29] Deharo E., Bourdy G., Quenevo C., Muñoz V., Ruiz G., Sauvain M. (2001). A search for natural bioactive compounds in Bolivia through a multidisciplinary approach. Part V. Evaluation of the antimalarial activity of plants used by the Tacana Indians. *Journal of Ethnopharmacology*.

[30] Geremedhin G., Bisrat D., Asres K. (2014). Isolation, characterization and in vivo antimalarial evaluation of anthrones from the leaf latex of *Aloe percrassa* Todaro. *Journal of Natural Remedies*.

[31] Fentahun S., Makonnen E., Awas T., Giday M. (2017). In vivo antimalarial activity of crude extracts and solvent fractions of leaves of *Strychnos mitis* in *Plasmodium berghei* infected mice. *BMC Complementary and Alternative Medicine*.

[32] Tarh J., Iroegbu C. (2017). In-vitro anti-bacterial activity of extracts of *Euphorbia abyssinica* (Desert Candle) stem-bark and latex. *Journal of Advances in Microbiology*.

[33] Eyasu M., Shibeshi W., Giday M. (2013). In vivo antimalarial activity of hydromethanolic leaf extract of *Calpurnia aurea* (Fabaceae) in mice infected with chloroquine sensitive *Plasmodium berghei*. *International Journal of Pharmacology*.

[34] Birru E. M., Geta M., Gurmu A. E. (2017). Antiplasmodial activity of Indigofera spicata root extract against *Plasmodium berghei* infection in mice. *Malaria Journal*.

[35] Fidock D. A., Rosenthal P. J., Croft S. L., Brun R., Nwaka S. (2004). Antimalarial drug discovery: efficacy models for compound screening. *Nature Reviews Drug Discovery*.

[36] Yang Z.-G., Sun H.-X., Fang W.-H. (2005). Haemolytic activities and adjuvant effect of *Astragalus membranaceus* saponins (AMS) on the immune responses to ovalbumin in mice. *Vaccine*.

[37] Mekonnen L. B. (2015). In vivo antimalarial activity of the crude root and fruit extracts of *Croton macrostachyus* (Euphorbiaceae) against *Plasmodium berghei* in mice. *Journal of Traditional and Complementary Medicine*.

[38] Teka T., Awgichew T., Kassahun H. (2020). Antimalarial activity of the leaf latex of *Aloe weloensis* (Aloaceae) against *Plasmodium berghei* in mice. *Journal of Tropical Medicine*.

